# Air-mode photonic crystal ring resonator on silicon-on-insulator

**DOI:** 10.1038/srep19999

**Published:** 2016-01-28

**Authors:** Ge Gao, Yong Zhang, He Zhang, Yi Wang, Qingzhong Huang, Jinsong Xia

**Affiliations:** 1 Wuhan National Laboratory for Optoelectronics, Huazhong University of Science and Technology, Wuhan, 430074, China; 2 State Key Laboratory of Advanced Optical Communication Systems and Networks, Department of Electronic Engineering, Shanghai Jiao Tong University, Shanghai, 200240, China

## Abstract

In this report, we propose and demonstrate an air-mode photonic crystal ring resonator (PhCRR) on silicon-on-insulator platform. Air mode is utilized to confine the optical field into photonic crystal (PhC) air holes, which is confirmed by the three-dimensional finite-difference time-domain simulation. PhCRR structure is employed to enhance the light-matter interaction through combining the whispering-gallery mode resonance of ring resonator with the slow-light effect in PhC waveguide. In the simulated and measured transmission spectra of air-mode PhCRR, nonuniform free spectral ranges are observed near the Brillouin zone edge of PhC, indicating the presence of the slow-light effect. A maximum group index of 27.3 and a highest quality factor of 14600 are experimentally obtained near the band edge. Benefiting from the strong optical confinement in the PhC holes and enhanced light-matter interaction in the resonator, the demonstrated air-mode PhCRR is expected to have potential applications in refractive index sensing, on-chip light emitting and nonlinear optics by integration with functional materials.

Optical microcavities, including microring resonators^1^, photonic crystal (PhC) cavities^2^,^3^,^4^ and microdisks^5^, are the key components for on-chip photonic integrated circuits. Due to the high quality factor (Q factor), small mode volume and compact device footprint, such microcavities are ideally suited for applications in lasers^6^, modulators^7^,^8^, filters^9^, sensors^10^, optical switches^11^, nonlinear optics^12^,^13^ and more.

Recently, a hybrid resonator structure, incorporating PhC holes into a standard microring resonator, was proposed and demonstrated^14^,^15^. The photonic crystal ring resonator (PhCRR) combines the whispering-gallery mode (WGM) resonance of microring resonator with the slow-light effect presented in PhC waveguide. When the resonant modes are near the Brillouin zone edge, the free spectral range (FSR) is decreasing and nonuniform due to the increase of group index^16^. Using this decreasing FSR induced by the slow-light effect, the footprint of the microring resonator can be reduced while preserving suitably small FSR to support multiple optical channels in wavelength-division multiplexing technology^17^. More importantly, the slow-light effect increases the Q factor of the resonant modes and enhances the light-matter interaction in PhCRR^14^,^18^. The increase in Q factor benefits from the slow-light dispersion near the band edge^19^, which results in the spectral narrowing of the resonance linewidth. The narrow spectral linewidth and strong light-matter interaction make the PhCRR a great choice for high-sensitive sensors^20^ and low-threshold lasers^15^.

Previous reports mainly concentrate on the dielectric-mode PhCRR. In dielectric mode, optical energy is predominantly confined in the higher-index material (dielectric region). Apparently, for biosensor applications, such mode profile is not advantageous because of the insufficient overlap between the optical field and the analyte inside the lower-index PhC holes. Here, to overcome this limitation, an air-mode PhCRR is proposed. The air-mode PhCRR shows the slow-light effect near the air band edge. Distinct from the dielectric-mode PhCRR, optical field is strongly squeezed into the PhC holes and overlaps sufficiently with the analyte in the air-mode PhCRR, which further improves the sensitivity of PhCRR structure. If the PhC holes are filled with light-emitting materials or nonlinear materials, the air-mode PhCRR is also promising for the realization of on-chip light emitters^21^ or nonlinear optical devices^22^. In this letter, we experimentally demonstrate the air-mode PhCRR. A highest Q factor of 14600 and a maximum group index of 27.3 are obtained near the Brillouin zone edge, which indicates that strong slow-light effect occurs in the air-mode PhCRR. The experimental results and strong optical confinement in PhC air holes are confirmed by the three-dimensional finite-difference time-domain (3D FDTD) simulation.

## Results and Discussion

### Device structure

**Figure 1:**
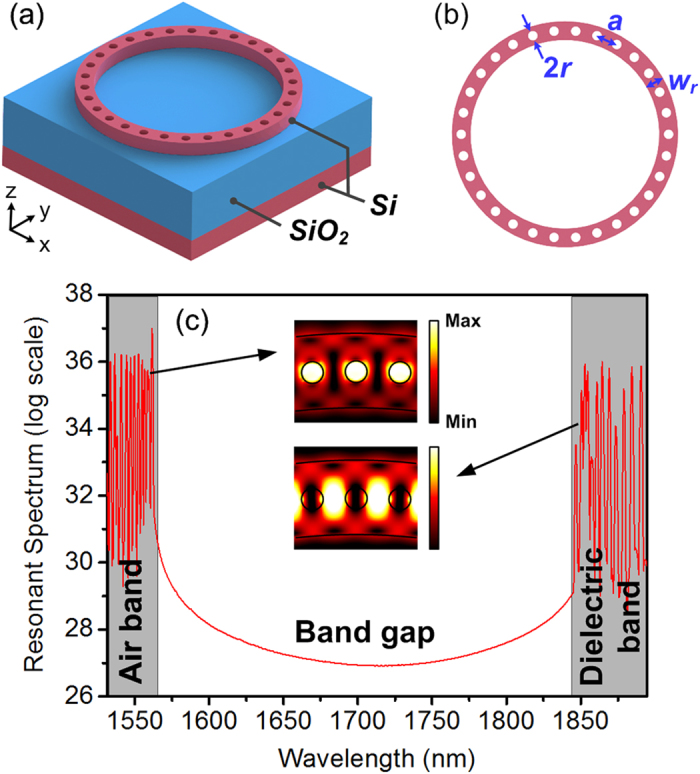
(**a**) Schematic diagram of the proposed air-mode PhCRR. (**b**) Geometrical parameters of the PhCRR structure. (**c**) Simulated resonant spectrum of the PhCRR by 3D FDTD. The simulated electric field (E-field) amplitude profiles of the resonant modes in the air band and dielectric band are shown in the insets.

The proposed device is based on a silicon-on-insulator (SOI) platform with a 220-nm-thick Si device layer and a 3-μm-thick buried oxide layer. Schematic diagram of the proposed air-mode PhCRR is shown in Fig. 1(a). Unlike the air-membrane PhC devices^23^, the underlying buried oxide layer is retained to provide mechanically stable support for PhCRR structure. From an optical perspective, however, the silica light line, which is lower than that of air, limits the available operating bandwidth for the air mode. In the previously studied dielectric-mode PhCRRs^16^, few of the wavevectors of the air mode is below the silica light line and the air band is in close proximity to the intrinsic absorption edge of Si. In the proposed air-mode PhCRR, the geometry structure is designed to enlarge the operating bandwidth for the air mode and place the air band at the telecom wavelength. The geometrical parameters of PhCRR, including ring width w_r_, lattice constant a and circular air-hole radius r, are defined in Fig. 1(b). With w_r_ = 680 nm, a = 430 nm, r = 0.24a, and total air-hole numbers of 240, the simulated resonant spectrum of the air-mode PhCRR is shown in Fig. 1(c). In 3D FDTD simulation, a TE-like polarized light excited by a dipole source in the ring resonator propagates through the PhCRR and then is monitored on the other side. Wavelength ranging from 1563 to 1845 nm corresponds to the photonic bandgap (PBG) region. The resonant peaks on two sides of the PBG region are located in the air band and dielectric band, respectively. Close to the Brillouin zone edge, the resonant modes in the two bands both exhibit nonuniform FSRs, indicating the presence of the slow-light effect. The simulated band-edge resonant mode profiles are shown in the insets of Fig. 1(c). As seen, optical field of the dielectric-band resonant mode is mainly localized in the dielectric region, while that of the air-band resonant mode has a good field overlapping with the PhC air holes.

**Figure 2:**
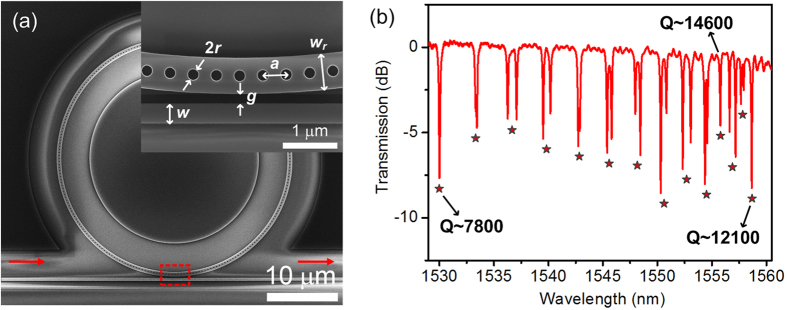
SEM image and measured transmission spectrum. (**a**) SEM image of the fabricated air-mode PhCRR. The inset shows a close-up of the evanescent coupling region. (**b**) Measured TE-polarized transmission spectrum of PhCRR. The resonances (or centers of resonance splitting) are identified with asterisks.

Figure 2(a) shows the scanning electron microscope (SEM) image of a fabricated air-mode PhCRR device. A strip waveguide is used to couple light into the PhCRR. The coupling efficiency depends on the phase matching and spatial overlapping between the guided mode of the strip waveguide and the resonant mode of the PhCRR^24^. Hence, to achieve effective coupling between the strip waveguide and ring waveguide with PhC air holes, the width of the strip waveguide is reduced with respect to that of the ring waveguide. A close-up of the evanescent coupling region is shown in the inset of Fig. 2(a). The gap separation g is 170 nm and the width of the strip waveguide w is 375 nm. The other geometrical parameters are identical to those used in the simulation.

### Experimental measurement

Figure 2(b) shows the measured optical transmission spectrum of the fabricated PhCRR device. Multiple resonant dips are observed in the wavelength range from 1530 to 1560 nm. The mode splitting at some of the resonances result from the strong mutual coupling between the two counter-propagating degenerate modes inside the PhCRR^24^. As resonant wavelength approaches the band edge, the FSRs decrease from 3.38 to 0.86 nm, indicating a strong dispersion of group index. The strong dispersion indicates that the slow-light effect occurs near the Brillouin zone edge of the one-dimensional PhC. It is worth noting that the slow-light effect increases the Q factor of the resonant modes. The Q factor at the resonance 1558.6 nm nearest the band edge is 12100, which is significantly higher than that (~7800) at the resonance 1530.0 nm away from the band edge. And the highest Q factor of 14600 is obtained at the resonance 1555.8 nm near the band edge. This is most likely because the Q-enhancement induced by dispersion overcomes the scattering loss due to the rough side walls of PhC holes. Note that a high Q factor is very important for refractive index (RI) sensing applications. A higher Q factor corresponds to a narrower spectral linewidth and a longer interaction time between the optical field and analyte, which can lead to a smaller limit of detection and a larger RI sensitivity. For the light emitters and nonlinear optical devices, a higher Q factor is also desirable to realize a larger Purcell factor and a longer effective nonlinear interaction length. To the best of our knowledge, our air-mode PhCRR exhibits a higher Q factor with respect to the dielectric-mode PhCRRs reported previously^16^,^20^. We attribute it to the smaller fluctuation of PhC-hole radii, reduced roughness of inner walls and larger microring radius.

### FDTD simulation

**Figure 3:**
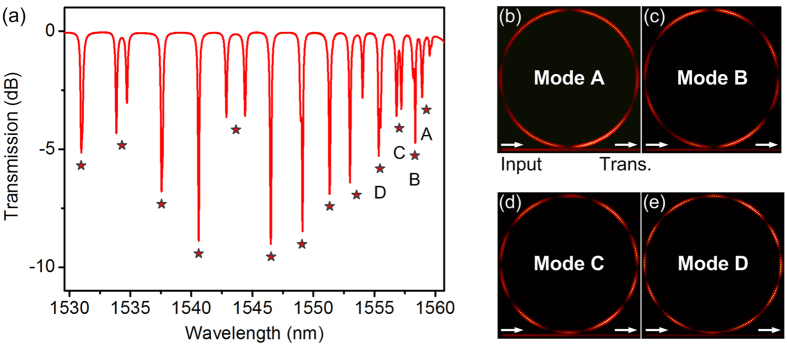
Simulated transmission spectrum and E-field distributions. (**a**) 3D FDTD simulated TE-polarized transmission spectrum of the air-mode PhCRR. (**b**–**e**) Simulated E-field distributions of the slow-light resonant modes A–D.

The 3D FDTD method is utilized to get more insight on the slow-light resonant modes of the air-mode PhCRR. The geometrical parameters are extracted from the SEM image. In simulation, a light source with a spatial shape as the fundamental TE mode is placed at the input port of the strip waveguide. Figure 3(a) shows the simulated transmission spectrum at the through port of the strip waveguide. When approaching the Brillouin zone edge, the decrease in FSRs from 3.31 to 0.95 nm indicates the large group index dispersion and strong slow-light effect. Comparing the transmission spectra in Figs 2(b) and 3(a), we find that there is a good agreement between the simulated and experimentally measured FSRs. The minor difference on extinction ratio (ER) is likely due to the low mesh accuracy in the evanescent coupling region in simulation, limited by our computing resources.

We label the first few slow-light resonant modes near the band edge and compute their corresponding steady-state E-field distributions as shown in Fig. 3(b–e). All the resonant modes show a standing wave pattern with an even number of nodes along the ring. The spatial beating results from a linear combination of two frequency-degenerate modes^18^.

### Comparison between the air mode and dielectric mode

**Figure 4:**
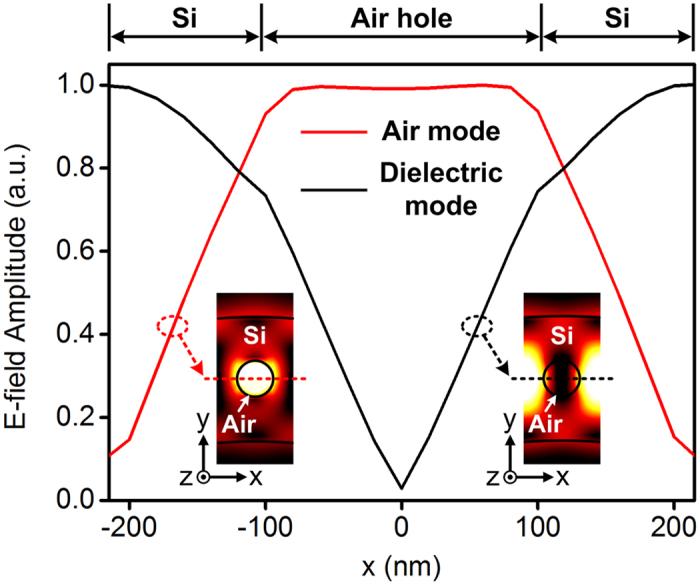
Comparison between the simulated E-field amplitude profiles of the air mode and dielectric mode along the x direction in one PhC unit cell. The insets show the simulated E-field distributions in the xy-plane.

In the bright high-field regions of all the simulated mode profiles, we observe that the E-field is strongly localized in the PhC air holes. In order to investigate this unique advantage of the air mode more carefully, a comparison between the E-field distributions of the air mode (mode D) and dielectric mode (band-edge resonant mode) is performed for one PhC unit cell in the high-field region, as shown in Fig. 4. The insets in Fig. 4 show the E-field distributions in the xy-plane at z = 0. Note that the origin of the coordinate system is set at the center of the air hole. The bright regions in the insets indicating a strong E-field are located in the air hole and silicon waveguide for the air mode and dielectric mode, respectively. The amplitude profiles of the E-field along the x cutline at y = 0 (as indicated by the dashed lines) are plotted for a quantitative observation. In the air (dielectric) mode, the maximum and minimum E-field amplitude are obtained in the air hole (silicon waveguide) and silicon waveguide (air hole), respectively. In the whole unit cell, ratio of the squared amplitude of the electric field in the air hole is about 27.5% in the air mode, which is significantly higher than that (4.3%) in the dielectric mode and comparable to the field confinement ratio in the air slot region of silicon slot waveguides^25^,^26^. Hence, the E-field distribution of the air mode is attractive to enable an effective interaction with integrated functional materials. In the fabricated air-mode PhCRR, the air-hole radius r = 103 nm is large enough to be filled with the analytes, light-emitting materials or nonlinear materials.

### Group index

**Figure 5:**
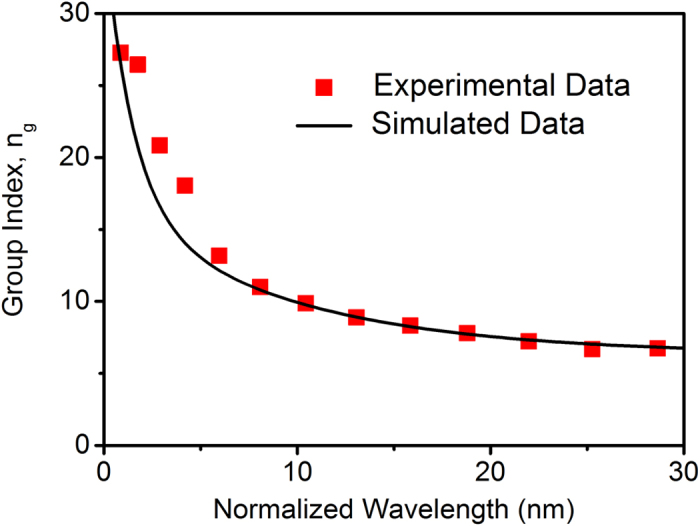
Measured and simulated group index. Group index calculated from the measured transmission spectrum (symbols) and fitting of group index calculated from the simulated transmission spectrum (curve).

From the above measured and simulated transmission spectra of the air-mode PhCRR in Fig. 2(b) and 3(a), the group index can be calculated by taking n_g_ ≈ λ^2^/(FSR·L), where n_g_ is the group index, λ is the central wavelength between two adjacent resonances, and L is the round-trip path-length of the ring. Note that in the presence of resonance splitting, the center of splitting is used to figure out the value of FSR. Figure 5 plots the group index calculated from the measured and simulated transmission spectra as a function of wavelength. Here, the wavelength is normalized to the resonance wavelength nearest the band edge for a better observation of the increasing trend of group index as the resonances approach the band edge. The group index calculated from the simulated transmission spectrum is fitted for comparison with the measured value. Good agreement exists between the experimental and simulated results. The largest measured group index of the air-mode PhCRR is about 27.3. And the corresponding slowdown factor S ≈ 8 is obtained by taking S = n_g_/n_p_^27^, where n_p_ is the phase index of silicon. For the resonance with the highest Q factor in Fig. 2(b), the calculated group index is about 20.8 and the corresponding slowdown factor is 6. The strong slow-light effect can lead to the compression of optical energy in space, enhancing light-matter interaction. Moreover, by carefully designing the periodic waveguide in the air-mode PhCRR, the group index is expected to be further increased. Specifically, by adjusting the filling fraction r/a or the ring width w_r_, the shape of the dispersion relation of the periodic waveguide can be varied^18^,^28^, enabling the tuning of group index.

In summary, we have proposed and demonstrated an air-mode PhCRR on silicon-on-insulator. In such type of PhCRR, optical field can be strongly confined in PhC air holes, proven by the 3D FDTD simulated band-edge resonant mode profiles. As wavelength approaches the Brillouin zone edge, the transmission spectrum of the PhCRR exhibits a decreasing FSR, indicating the presence of the slow-light effect. A maximum group index of 27.3 (corresponding to a slowdown factor of 8) and a highest Q factor of 14600 are experimentally obtained near the band edge. The combination of the strong optical confinement in air holes, the slow-light effect and the WGM resonance would greatly enhance the light-matter interaction in air holes, which makes the air-mode PhCRR potential for applications in RI sensing, on-chip light emitting and nonlinear optics.

## Methods

### Device fabrication

The device is fabricated on a commercial SOI wafer (SOITEC). E-beam lithography (Vistec EBPG5000 Plus) is used to define the air-mode PhCRR structure on the ZEP520A resist. Then the pattern is transferred to the top silicon layer by inductively coupled plasma dry etching using SF_6_ and C_4_F_8_ gases (Oxford Instruments Plasmalab System100).

### Transmission spectrum measurement

The fabricated PhCRR device is characterized by an optical transmission measurement. TE-polarization light from a broadband amplified spontaneous emission (ASE) source is coupled into the strip waveguide using grating coupler and then coupled into the PhCRR through evanescent-field coupling. The light at the through port of the strip waveguide is coupled out into a fiber using another grating coupler and then measured by an optical spectrum analyzer.

## Additional Information

**How to cite this article**: Gao, G. *et al*. Air-mode photonic crystal ring resonator on silicon-on-insulator. *Sci. Rep.* **6**, 19999; doi: 10.1038/srep19999 (2016).
